# Medical Professional Enhancement Using Explainable Artificial Intelligence in Fetal Cardiac Ultrasound Screening

**DOI:** 10.3390/biomedicines10030551

**Published:** 2022-02-25

**Authors:** Akira Sakai, Masaaki Komatsu, Reina Komatsu, Ryu Matsuoka, Suguru Yasutomi, Ai Dozen, Kanto Shozu, Tatsuya Arakaki, Hidenori Machino, Ken Asada, Syuzo Kaneko, Akihiko Sekizawa, Ryuji Hamamoto

**Affiliations:** 1 Artificial Intelligence Laboratory, Research Unit, Fujitsu Research, Fujitsu Ltd., 4-1-1 Kamikodanaka, Nakahara-ku, Kawasaki 211-8588, Japan; akira.sakai@fujitsu.com (A.S.); yasutomi.suguru@fujitsu.com (S.Y.); 2 RIKEN AIP-Fujitsu Collaboration Center, RIKEN Center for Advanced Intelligence Project, 1-4-1 Nihonbashi, Chuo-ku, Tokyo 103-0027, Japan; rkomatsu@med.showa-u.ac.jp (R.K.); ryu@med.showa-u.ac.jp (R.M.); 3 Department of NCC Cancer Science, Biomedical Science and Engineering Track, Graduate School of Medical and Dental Sciences, Tokyo Medical and Dental University, 1-5-45 Yushima, Bunkyo-ku, Tokyo 113-8510, Japan; 4 Division of Medical AI Research and Development, National Cancer Center Research Institute, 5-1-1 Tsukiji, Chuo-ku, Tokyo 104-0045, Japan; adozen@ncc.go.jp (A.D.); kshozu@ncc.go.jp (K.S.); hidenori.machino@riken.jp (H.M.); ken.asada@riken.jp (K.A.); sykaneko@ncc.go.jp (S.K.); 5 Cancer Translational Research Team, RIKEN Center for Advanced Intelligence Project, 1-4-1 Nihonbashi, Chuo-ku, Tokyo 103-0027, Japan; 6 Department of Obstetrics and Gynecology, School of Medicine, Showa University, 1-5-8 Hatanodai, Shinagawa-ku, Tokyo 142-8666, Japan; arakakit@med.showa-u.ac.jp (T.A.); sekizawa@med.showa-u.ac.jp (A.S.)

**Keywords:** explainable artificial intelligence, deep learning, fetal cardiac ultrasound screening, congenital heart disease, abnormality detection

## Abstract

Diagnostic support tools based on artificial intelligence (AI) have exhibited high performance in various medical fields. However, their clinical application remains challenging because of the lack of explanatory power in AI decisions (black box problem), making it difficult to build trust with medical professionals. Nevertheless, visualizing the internal representation of deep neural networks will increase explanatory power and improve the confidence of medical professionals in AI decisions. We propose a novel deep learning-based explainable representation “graph chart diagram” to support fetal cardiac ultrasound screening, which has low detection rates of congenital heart diseases due to the difficulty in mastering the technique. Screening performance improves using this representation from 0.966 to 0.975 for experts, 0.829 to 0.890 for fellows, and 0.616 to 0.748 for residents in the arithmetic mean of area under the curve of a receiver operating characteristic curve. This is the first demonstration wherein examiners used deep learning-based explainable representation to improve the performance of fetal cardiac ultrasound screening, highlighting the potential of explainable AI to augment examiner capabilities.

## 1. Introduction

With the rapid development of tools to support medical diagnosis using artificial intelligence (AI), expectations from AI have been increasing continuously [1,2,3,4,5]. However, in reality, the application of AI in clinical practice remains challenging. One of the major obstacles is regarded as the “black box problem” of AI [4,6,7,8]. The black box problem is a problem in which the relationship between input and output obtained from data is so complicated that any human, including the developer, cannot determine the rationale for the AI decision [9]. There are three major approaches for achieving explainable AI using a deep neural network (DNN), a machine learning technology typically used in medical imaging for diagnosis support. The first is a method for visualizing or analyzing the internal behavior of existing high-performance DNNs [10,11,12,13,14]. The second is to add an explanatory module to a DNN externally [12,15,16,17,18,19]. The third is to make DNNs perform decisions via explainable representations, which is also called “interpretable models” [20,21,22,23,24]. Of these, the third approach is the best in terms of achieving a high-level explanatory power. However, the first and second approaches have traditionally been actively pursued in explainable AI studies because interpretable models may cause performance degradation.

In the present study, we employ the third approach, i.e., interpretable models. One reason for its choice is that the performance of conventional AI is already high, and we can accept slight performance degradation. The second reason is that our purpose of developing AI diagnostic imaging support technology is not to improve the performance of the technology alone, rather to enhance the performance of medical professionals using this technology. A more sophisticated explainable representation has the potential to enhance the performance of medical professionals. Therefore, we propose a novel interpretable model targeting videos of fetal cardiac ultrasound screening, one of the crucial obstetric examinations; however, its detection rate of congenital heart diseases (CHDs) remains low [25,26,27]. This interpretable model is an auto-encoder that includes two novel techniques, cascade graph encoder and view-proxy loss, and generates a “graph chart diagram” as an explainable representation. The graph chart diagram visualizes the detection of substructures of the heart and vessels in the screening video on a two-dimensional trajectory and, thereafter, calculates the abnormality score by measuring the deviation from the normal. The examiner uses the graph chart diagram and abnormality score to perform fetal cardiac ultrasound screening.

However, studies on the comparison or collaboration between AI and humans are vital to obtain insight into the clinical implementation of AI, and many studies have been conducted in this regard [12,28,29,30,31,32,33] with several of them on ultrasound [34,35,36,37,38]. Improvement in the performance of combining human and AI scores has also been studied in the field of dermatology [39], breast oncology [40], and pathology [41,42,43]. A small number of studies have reported the performance of examiners actually using AI [44]. Regarding the use of explainable AI, Yamamoto et al. used explainable AI to gain new insights into pathology [42]. Tschandl et al. [44] educated medical students about insights obtained from Grad-CAM [10]. However, to the best of our knowledge, there is no study in which examiners directly utilized deep learning-based explainable representations (e.g., heatmap, compressed representation, and graph) in the field of medical AI. We believe the reason for this is that the current mainstream techniques have low consistency between decisions and explanations [4,20]. Because decisions (or AI score) and explanations are generated from the same process in interpretable models, consistency between explanations and decisions is high, and performance enhancement by adding explanations to decisions is most expected [20].

In this study, we attempted to verify whether the deep learning-based explanatory representation “graph chart diagram” could enhance the detection of CHD anomalies for 27 examiners (8 experts, 10 fellows, and 9 residents). Quantitative evaluation using the arithmetic mean of the area under the curve (AUC) of the receiver operating characteristic (ROC) curve showed that the screening performance was improved by utilizing the graph chart diagram in all groups: expert, fellow, and resident groups. This is the first report to demonstrate improved screening performance for CHD using explainable AI, and it presents a new direction for the introduction of explainable AI into medical testing and diagnosis.

## 2. Materials and Methods

### 2.1. Data Preparation

The total dataset used in this study consists of 160 cases and 344 videos (18–34 weeks gestation). We used 13 CHD cases and 26 videos as the abnormal data, which has been confirmed by postnatal testing. We used only normal data from 134 normal cases (292 videos) to train DNNs explained in Section 2.2; 108 cases (247 videos) for object detection model YOLOv2 [45]; 60 cases (151 videos) for training the proposed auto-encoders. Referring to previous studies [46,47,48], the image number of our training data is in the same order of data size as the MNIST dataset, which was sufficient to achieve enough performance in this study. The validation set consisted of three normal cases and six videos for the normal data and three CHD cases and six videos for the abnormal data. In contrast, 10 cases and 20 videos of the normal data and 10 CHD cases and 20 videos of the abnormal data were used for the test dataset. Details of the CHD cases of the abnormal data are shown in Supplementary Table S1. The splitting ratio of the data was standard for machine learning [47,48,49]. No cases in the validation and test set overlapped with the dataset for training DNNs. All videos were taken by scanning the probe from the abdominal view to 3VTV (three-vessel trachea view) via 4CV (four-chamber view). All data were acquired using the Voluson^®^ E8 or E10 ultrasound machine (GE Healthcare, Chicago, IL, USA) at the four Showa University Hospitals (Tokyo and Yokohama, Japan) in an opt-out manner. The probe was an abdominal 2–6 MHz transducer, and a cardiac preset was used.

### 2.2. Proposed Method

**Figure 1:**
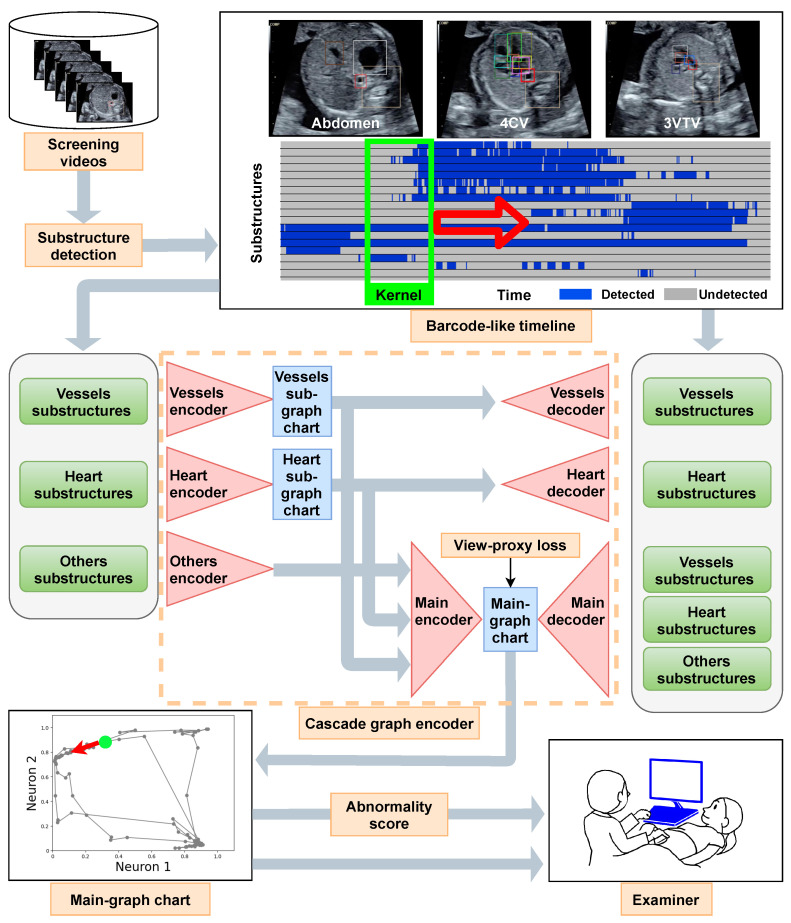
Flow chart of the proposed method. The screening videos were scanned in one direction from the stomach to the head. Therefore, a video contains diagnostic planes from the abdomen to the four-chamber view (4CV) and three-vessel trachea view (3VTV). The barcode-like timeline represents the detection of 18 substructures $R_{\mathrm{all}}$ for each frame of the screening video arranged in the time direction. The kernel slices the barcode-like timeline and feeds it to the cascade graph encoder. The kernel moves in the time direction (red open arrow). A sub-graph chart diagram was created for each of the four vessels $R_{\mathrm{vessels}}$ and the eight-heart substructures $R_{\mathrm{heart}}$. Information on the six other substructures $R_{\mathrm{others}}$ ( $=R_{\mathrm{all}}\backslash (R_{\mathrm{vessels}}\cup R_{\mathrm{heart}}$) is appended to two sub-graph chart diagrams to obtain the main graph chart diagrams. The view-proxy loss is applied to the main-graph chart diagram so that the main graph chart diagram is generated stably. The gray circle dots in the main-graph chart correspond to the parts of the barcode-like timeline that was sliced into the kernel at a certain time. In particular, the green dot indicates the point corresponding to the green kernel and moves in the direction of the red arrow. An abnormality score is calculated using the main-graph chart diagram and provided to the examiner. $R_{\mathrm{all}}$ is the set of { crux, ventricular septum, right atrium, tricuspid valve, right ventricle, left atrium, mitral valve, left ventricle, pulmonary artery, ascending aorta, superior vena cava, descending aorta, stomach, spine, umbilical vein, inferior vena cava, pulmonary vein, ductus arteriosus}. $R_{\mathrm{vessels}}$ is the set of the {pulmonary artery, ascending aorta, superior vena cava, ductus arteriosus} $R_{\mathrm{heart}}$ is the set of {crux, ventricular septum, right atrium, tricuspid valve, right ventricle, left atrium, mitral valve, left ventricle}. $R_{\mathrm{others}}$ set of $R_{\mathrm{all}}\backslash \left(R_{\mathrm{vessels}}\cup R_{\mathrm{heart}}\right)$.

Figure 1 shows a schematic flow of the proposed method, which is explained below. Firstly, we describe the proposed explainable representation, i.e., graph chart diagram. Subsequently, we describe two techniques to obtain a better graph chart diagram. The formula for graph chart diagram and two techniques is also provided in Appendix A. Finally, we explain how to calculate the abnormality score.

#### 2.2.1. Graph Chart Diagram

Komatsu et al. [46] showed that CHDs can be detected with high performance by annotating the substructures of normal hearts and vessels and deeming them abnormal if normal substructures are not found in the frame where they should be. They also proposed a “ barcode-like timeline”, which is a table indicating the substructure detection status for each frame of an ultrasound video scanned from the stomach to the heart. In the barcode-like timeline, the substructure is on the vertical axis and the time is on the horizontal axis. The substructures used in the barcode-like timeline are $R_{\mathrm{all}}=\{$crux, ventricular septum, right atrium, tricuspid valve, right ventricle, left atrium, mitral valve, left ventricle, pulmonary artery, ascending aorta, superior vena cava, descending aorta, stomach, spine, umbilical vein, inferior vena cava, pulmonary vein, ductus arteriosus}. To detect abnormalities, examiners must specify the location of the diagnostic plane; however, fetal movement and inappropriate probe movement make it difficult.

We can address this problem of barcode-like timelines using graph chart diagrams. A graph chart diagram represents the substructure detection status converted from a barcode-like timeline into a trajectory of points in two dimensions using an auto-encoder with two neurons in the intermediate layer (X and Y axes denote the outputs of neurons 1 and 2, respectively). The trajectory on the graph chart diagram is expected to have a constant shape regardless of the probe movement if it is not an abnormal video. The examiner can determine whether the patient has CHD by assessing the deviation of the shape from the normal one, without specifying the diagnostic plane. The computational time to create and display a graph chart diagram is real time. For more details, Appendix A.1 elaborates on the graph chart diagrams.

#### 2.2.2. View-Proxy Loss and Cascade Graph Encoder

A simple auto-encoder can form graph chart diagrams by learning from the normal data; however, we propose view-proxy loss and a cascade graph encoder to improve graph chart diagrams for practical applications. The view-proxy loss prevents learning instability due to the “entanglement” of graph chart diagrams. The cascade graph encoder improves explainability by creating sub-graph chart diagrams for some group substructures and then creating a main-graph chart diagram based on them.

**Figure 2:**
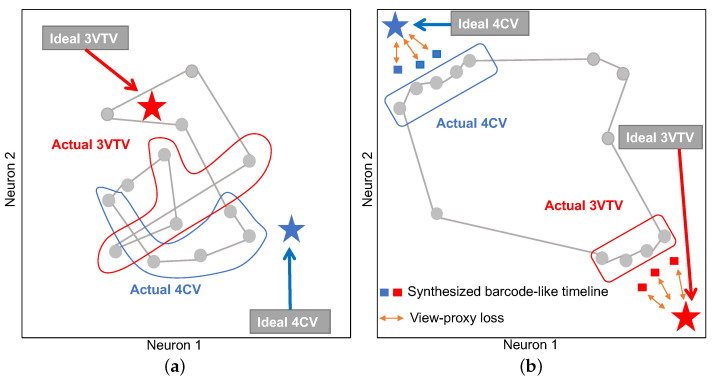
Schematic diagram of the effect of view-proxy loss. (**a**) Graph chart diagram without view-proxy loss. The graph chart diagram (grey connected circles) without view-proxy loss is formed, in which points corresponding to actual 4CV (surrounded by the blue line), actual 3VTV (surrounded by the red line), and other planes are randomly mixed. (**b**) Graph chart diagram with view-proxy loss. The stars denote the coordinates for the ideal 4CV (blue) and 3VTV (red). The squares denote corresponding points to the synthesized barcode-like timelines for 4CV (blue) and 3VTV (red). The view-proxy loss (orange bidirectional arrows) considers the loss between the stars and squares. As a corollary of optimizing the view-proxy loss, the grey points surrounded by the blue or red lines are attracted to their respective specified coordinates. As a result of optimizing the view-proxy loss, the graph chart diagram is expected to be untangled.

Figure 2 illustrates a schematic diagram of the effect of the view-proxy loss. Graph chart diagrams generated by a simple auto-encoder have no “guideline” for determining the formation, and thus tend to form a tangled shape, as shown in Figure 2a. The purpose of optimizing the view-proxy loss is to attract points on the graph chart diagram corresponding to the typical diagnostic planes 3VTV and 4CV to the target coordinates (1,0) and (0,1), respectively. We cannot directly attract the points on the graph chart diagram corresponding to the diagnostic planes to the target coordinates because videos do not have annotations of diagnostic planes. Therefore, the proposed method synthesizes the ideal barcode-like timeline data corresponding to the normal diagnostic planes (i.e., 3VTV and 4CV) and takes the distance between the corresponding point and the target point, which is the view-proxy loss (Figure 2b). By minimizing this loss, we can attract points on the actual graph chart diagram as a collateral effect. For more details, Appendix A.2 elaborates on the view-proxy loss.

The cascade graph encoder (Figure 1) constructs sub-graph chart diagrams as a further source of explanatory information. Concretely, we creates a sub-graph chart diagram for the vessels $R_{\mathrm{vessels}}=$ {pulmonary artery, ascending aorta, superior vena cava, ductus arteriosus} and heart $R_{\mathrm{heart}}=$ {crux, ventricular septum, right atrium, tricuspid valve, right ventricle, left atrium, mitral valve, left ventricle}. Thereafter, we combine the information for the remaining the substructures with the two sub-graph chart diagrams to create the main-graph chart diagram, which is the most comprehensive representation. We apply the view-proxy loss only to the main-graph chart diagram. Moreover, Appendix A.3 details the cascade graph encoder.

#### 2.2.3. Abnormality Score

We need further processing to quantitatively evaluate the degree of abnormalities as a scalar number because the graph chart diagram is an explainable representation for visual assessment. If the video contains an abnormality, the area of the region drawn by the point on the graph chart diagram is smaller than that of the normal video. This is because the normal barcode-like timeline patterns are absent if there are abnormal substructures. Therefore, we can quantitatively evaluate the abnormality score of a graph chart diagram by calculating the area of the figure drawn by the trajectory of a point. We used the Shapely package in Python to create shapes from the trajectories of points and calculate their areas in this study. Thereafter, we calculated the abnormality score by normalizing it such that the score is 0 for the maximum area and 1 for zero area:

(1) $$\Gamma_{\mathrm{AI}}\left(G\right)=1-\frac{f_{\mathrm{area}}\left(G\right)}{f_{\mathrm{area}}\left(G_{\max}\right)}.$$

The target graph chart diagram is *G*, the function for calculating the area is $f_{\mathrm{area}}$, and the graph chart diagram with the largest area in the test data is $G_{\max}$.

### 2.3. Evaluation of Medical Professional Enhancement

We conducted a comparative study to evaluate the improvement in the screening performance of examiners resulting from the use of a graph chart diagram. There were 8 experts, 10 fellows, and 9 residents enrolled in this study. All the examiners belonged to Showa University Hospitals (Tokyo and Yokohama, Japan). The procedure of this test was based on a previous study [50]. The examiners rated each video of the test dataset (20 videos of 10 normal cases and 20 videos of 10 CHD cases; types of CHDs are provided in Supplementary Table S1), shown in the Methods section as normal or abnormal with five confidence levels. We consider *d* (−1 for normal and 1 for abnormal) as the decision and *c* (integer of 1–5) as the confidence level and calculate the abnormality score for each video using the following formula.

(2) $$\Gamma_{\mathrm{human}}\left(G\right)=\frac{dc+5}{10}.$$

The test procedure comprised an instruction part and two main blocks.

- Instruction part ⋯ The examiners were instructed on how to perform the test, and a graph chart diagram was explained. The examiners were given samples of the main-graph chart diagrams of a pair of normal and abnormal videos. We used a different model and different videos for testing to generate these main-graph chart diagrams. The performance of AI was not explained to examiners. Regarding the types of CHDs, the examiners were not informed what types of diseases would be included. Considering the ratio of normal to abnormal cases, we did not inform the examiners of the amounts of each.
- First block ⋯ The examiners were given 40 randomly numbered videos and an Excel file to fill in the answers. They played the videos on a laptop computer and filled in the Excel file with their decisions and confidence levels. No protocol was provided on how to assess the videos in detail to allow the examiners to perform this test as they usually would perform fetal cardiac ultrasound screening in a clinical setting. Therefore, the first block was performed depending on each examiner’s education and skill level.
- Second block ⋯ The examiners evaluated the same dataset independently of the first block, referring to graph chart diagrams *G* and abnormality scores $\Gamma_{\mathrm{AI}}\left(G\right)$ for each video. The graph chart diagrams were given as PNG files, and the anomaly scores were given in an Excel sheet. The decisions of the AI between the normal and abnormal cases were not presented. Shapes created by the Shapely package were also not provided to the examiners. Considering the choice of graph chart diagrams *G* and abnormality scores $\Gamma_{\mathrm{AI}}\left(G\right)$, we adopted the results with the third-best AUC of the ROC curve among the five trials.

The examiners had no time limit, and were allowed to change their decisions and confidence levels once they had made it within each block. The examiners spent 20 to 40 min to complete this test, and were not informed of their own results until every test was completed.

### 2.4. Statistical Analysis

All the numerical experiments were evaluated using the AUC of the ROC curve. The numerical experiments were run five times with different random seeds, and the mean, standard deviation, median, and maximum and minimum values of the AUC were reported. Considering the experiments in which examiners were enrolled, we calculated the accuracy, false-positive rate (FPR), precision, recall, and F1 scores for an abnormality score of 0.5 in addition to the AUC of the ROC curve.

## 3. Results

Firstly, we show examples of graph chart diagrams, which are the representation proposed in this study. Subsequently, we show the evaluation of abnormality detection performance of AI only by abnormality scores calculated from graph chart diagrams. Finally, we show the performance improvement when the examiners used the graph chart diagrams. Details of the numerical experiments are explained in Appendix B.

### 3.1. Examples of Graph Chart Diagram

**Figure 3:**
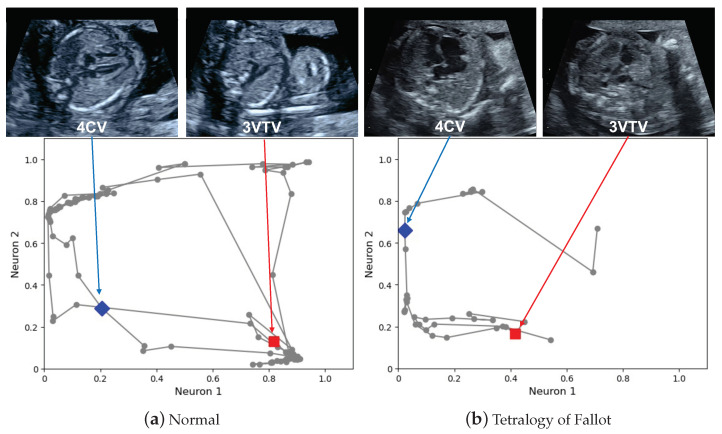
Main-graph chart diagrams. The main-graph chart diagrams were obtained from the fetal cardiac ultrasound screening video of a normal case (**a**) and tetralogy of Fallot case (**b**). The dots circled in gray correspond to each kernel, and these dots are connected as the kernel moves. Several points correspond to the typical diagnostic planes. The four-chamber view (4CV) and three-vessel trachea view (3VTV) are shown as blue diamond and red square points, respectively.

Figure 3 shows main-graph chart diagrams corresponding to a normal case and abnormal case of tetralogy of Fallot (TOF), respectively. The video corresponding to the normal case is provided in Supplementary Video S1, and that of the TOF case is provided in Supplementary Video S2. Examples of the created shapes corresponding to Figure 3 are provided in Supplementary Figure S1. Considering the normal video, the points are spread over the entire graph, and they are attracted to the (0,1) coordinate for the points corresponding to the heart-related planes and the (1,0) coordinate for the points corresponding to the large vessels-related planes. The attraction of these points to the particular coordinates is the effect of the view-proxy loss (Figure 2 shows the concept, and the mechanism is explained in Section 2.2.2). Regarding the TOF case, there is a large shift, especially in the points corresponding to the three-vessel trachea views (3VTV). The points do not pass through these areas because the auto-encoder does not recognize the pulmonary artery and other large vessels as normal. The area of the region drawn by the trajectory of the points becomes smaller in the graph chart diagram of the abnormal video.

**Figure 4:**
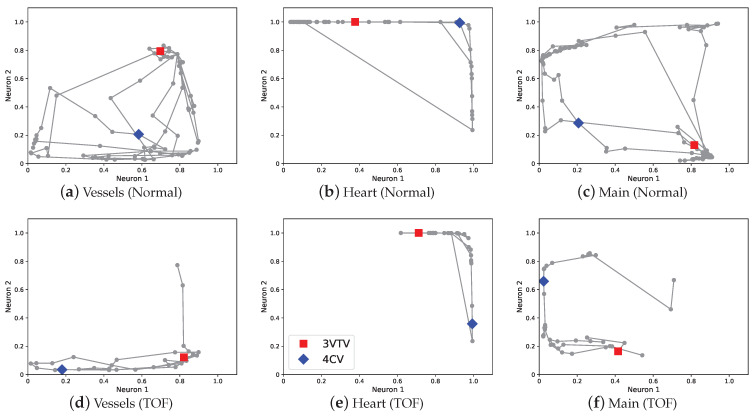
Sub-graph chart diagrams. (**a**,**b**) show sub-graph chart diagrams of the vessels and heart in the normal video, respectively. (**d**,**e**) show the sub-graph chart diagrams of the vessels and heart in the TOF video, respectively. (**c**,**f**) are the same as (**a**,**b**) in Figure 3, and are presented for reference. The blue diamond points correspond to the 4CV, and the red square points correspond to the 3VTV. TOF, tetralogy of Fallot; 4CV, four-chamber view; 3VTV, three-vessel trachea view.

The cascade graph encoder provides sub-graph chart diagrams as explained in Section 2.2.2. Figure 4 shows the sub-graph chart diagrams of the normal video and abnormal video of the TOF case. The sub-graph chart diagram of vessels compresses the information of $R_{\mathrm{vessels}}$ and the sub-graph chart diagram of the heart compresses the information of $R_{\mathrm{heart}}$. The sub-graph chart diagram of the abnormal video shows a decrease in the movement of points in both the vessels and heart. Particularly, the change in the sub-graph chart diagram of the vessels is large, and it is confirmed that the density of points in the region corresponding to 3VTV marked by red square decreased (Figure 4a,d). These deviations in the graph chart diagrams are consistent with the disease characteristic in TOF of abnormalities in the blood vessels of $R_{\mathrm{vessels}}$.

### 3.2. Screening Performance Using Only AI

As explained in Section 2.2.2, the proposed method has two techniques: view-proxy loss and a cascade graph encoder. We performed ablation tests on these two techniques to verify the performance of the proposed method. Five trials were run in the numerical experiments, and the initial values of network weights were initialized using random numbers of different seeds. A total of 40 videos of the test dataset were used to evaluate the performance, which consisted of 20 videos of 10 normal cases as normal data and 20 videos of 10 CHD cases as abnormal data (types of CHDs are provided in Supplementary Table S1). We evaluated the experiments using the mean AUC of the ROC curve.

Table 1 shows the results of the numerical experiments. The mean AUC of the ROC curve using a simple auto-encoder was 0.798, the standard deviation was 0.007, and the median was 0.803. The view-proxy loss increased the mean to 0.833 and decreased the standard deviation to 0.002, and the median increased to 0.833. The view-proxy loss was introduced to prevent the graph chart diagram from being different for each training by attracting points corresponding to 4CV and 3VTV to particular fixed coordinates. It did not only reduce the standard deviation but also contributed to the performance improvement. The cascade graph encoder also improved the performance, with a mean, standard deviation, and median of 0.819, 0.013, and 0.813, respectively. The idea of performing a dimensional compression in advance for each relevant substructure was confirmed to improve the performance. The combination of graph cascade encoder and view-proxy loss improved the performance with a mean, standard deviation, and median of 0.861, 0.003, and 0.860, respectively. The combination of these two techniques was successful in improving performance as well as stabilizing the training.

### 3.3. Screening Performance Enhancement Using the Examiner and AI Collaboration

**Figure 5:**
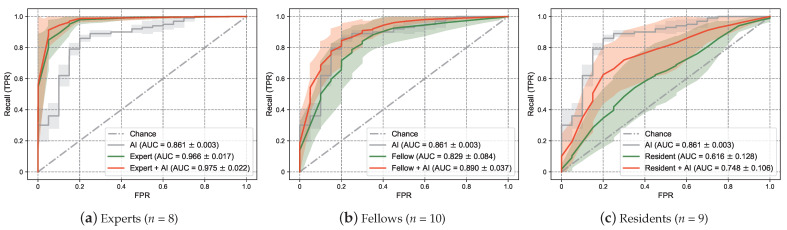
Performance of the AI-assisted fetal cardiac ultrasound screening. The ROC curves show the screening performance comparison of experts (**a**), fellows (**b**), and residents (**c**) with and without AI assistance. The color of the solid lines represents the ROC curve for AI only (gray), examiner only (green), and examiner with AI (red). Solid lines indicate mean values. Lighter semitransparent colors surrounding the the solid lines indicate the standard deviation. The mean and standard deviation of the AUC of the ROC curves are also reported in the legends. ROC, receiver operating characteristics; AUC, area under the curve; TPR, true positive rate; FPR, false-positive rate.

Figure 5 shows the ROC curve using the abnormality score calculated by $\Gamma_{\mathrm{human}}\left(G\right)$. Table 2 shows the AUC of the ROC curve. The performance of only AI showed that the mean AUC of the ROC curve was 0.861, which was higher than 0.829 of the mean AUC of the ROC curve for only the fellow. The performance of the experts was 0.966, which was higher than that of the AI only. The performance of the residents only was 0.616, which was lower than that of AI only. Regarding the examiner and AI collaboration, the AI assistance increased the performance for experts, fellows, and residents. The residents recorded the largest increase in performance with AI of 0.132, increasing from 0.616 to 0.748. We also evaluated the performance using several metrics by setting the threshold. We adopted an abnormality score of 0.5 as the threshold value, which is consistent with the actual decision *c* made by the examiners (Equation (2)). The result is shown in Table 3. The AI assistance improved the accuracy of all the examiners. The experts, fellows, and residents only had mean accuracies of 0.928, 0.775, and 0.603, respectively. The experts + AI, fellows + AI, and residents + AI had accuracies of 0.938, 0.823, and 0.731, respectively. The AI assistance also improved the F1 score for all the examiners. The experts, fellows, and residents only had F1 scores of 0.934, 0.774, and 0.589, respectively. In contrast, the expert + AI, fellow + AI, and resident + AI exhibited F1 scores of 0.943, 0.822, and 0.716, respectively. However, the trends in precision and recall depended on the examiner’s experience. The experts also increased their recall 0.007 (=0.963−0.956) and precision 0.015 (=0.927−0.912) using AI. Moreover, the fellows increased their recall 0.045 (=0.825−0.780) and precision 0.047 (=0.819−0.772) using AI. The residents also increased their recall 0.122 (=0.694−0.572) and precision 0.130  (=0.746−0.616) using AI. Therefore, experts tended to increase their precision more than recall using the AI assistance; however, the fellows and residents tended to increase both.

## 4. Discussion

In this study, we proposed a deep learning-based explainable representation (graph chart diagram) that compresses and represents the information in fetal cardiac ultrasound screening videos and introduced two factors to realize the depiction of the graph chart, including a cascade graph encoder and view-proxy loss. We also demonstrated that the graph chart diagram and the abnormality score could improve the ability of examiners to detect abnormalities.

Research on explainability in deep learning has concentrated on analyzing models [10,11,12,13,14] or developing external modules [12,15,16,17,18] for explainability. Limited research has been conducted on interpretable models that modify the structure of the model. Some interpretable models improve the explanatory power by replacing modules [21,24]; however, domain-specific methods are also not much studied [19,22] because of the need for domain-specific knowledge [20]. Furthermore, we discuss interpretable models in a broader context. Studies have been extensively conducted to obtain human-interpretable representations from highly complex data [51,52,53,54], and several of these have been on compressing time-series information to a lower dimensionality [55]. Considering the deep learning field, TimeCluster was proposed to reduce the dimensionality of time-series information with a kernel and to represent the time-series information with a two-dimensional diagram [56]. TimeCluster targets single and very long time-series information and finds anomalies in a part of it. Therefore, TimeCluster compresses the dimensions using autoencoders and applies principal component analysis [51] or other projection methods to the intermediate representation. TimeCluster learns the network weights for each instance of the time-series information; therefore, different representations can be obtained for the same data. This indicates that TimeCluster is not designed to process the time-series information from several inspection videos of approximately 10 s to identify anomalies in the entire video. Therefore, our proposed method for the graph chart diagram learns many instances of normal videos. The intermediate layer learns a two-dimensional representation directly and does not train any network weight on the test videos.

Subsequently, we discuss the two proposed techniques, view-proxy loss and a cascade graph encoder. The view-proxy loss improves performance (Table 1) and reduces standard deviation by fixing the coordinates where the ideal 4CV and 3VTV appear on the graph chart diagram (Figure 2). The view-proxy loss can be regarded as one of the proxy losses [57,58,59]. A proxy loss creates a proxy from the data belonging to one class and includes the loss between the proxy and other samples. The view-proxy loss assumes the point corresponding to the ideal diagnostic plane as the proxy and considers the loss between the proxy and the synthesized barcode-like timeline corresponding to 4CV and 3VTV. The view-proxy loss is unique because the ideal diagnostic plane is known and is utilized as a proxy, and it synthesizes barcode-like timelines to solve the problem that there is no annotation of 4CV or 3VTV. The cascade graph encoder improves performance (Table 1) and explainability by creating sub-graph chart diagrams of sets of substructures (Figure 4), followed by a main-graph chart diagram of all the sets. Although the cascade graph encoder is similar to the hierarchical auto-encoder [60] or stacked auto-encoder [61], it is unique because our graph chart diagrams comprise partial and comprehensive explanatory representations.

We analyze the qualitative features of the graph chart diagrams. The graph chart diagram discards unnecessary information in the ultrasound screening and emphasizes the necessary information. The backward and forward movements of the probe, the speed of the movement of the probe, and the movement of the fetus during video recording are not necessary information for fetal cardiac ultrasound screening. The shape does not change in the graph chart diagram, even if a phase similar to the one passed appears multiple times. This reduces the noise caused by fetal movement and probe movement. The spacing between the points also does not affect the shape. This reduces the effect of the speed at which the probe is moved. Thus, the graph chart diagram is robust to the intrinsic noise caused by probe movement. Furthermore, the graph chart diagram is helpful, considering explainability. Regarding a graph chart diagram, the coordinates corresponding to the plane of the normal structure are scattered over a two-dimensional diagram, which serves as a checkpoint. If the checkpoints cannot be seen in the video, a part of the shape will be missing. Therefore, the area of the shape functions as an indicator of the degree of abnormality. Regarding the detection of shapes, recognizing a shape from the trajectory of a point is an advanced technology. In Python, the Shapely package is a standard technology; nonetheless, a more advanced algorithm may improve the performance of the abnormality score $\Gamma_{\mathrm{AI}}$. Considering the experiment on collaboration between the examiners and AI, we provided raw point trajectories as shown in Figure 3 instead of the shapes shown in Supplementary Figure S1, because we expect the human shape recognition ability to outperform the algorithm.

Deep learning-based methods for automatically detecting diagnostic planes [62,63,64,65,66,67] and methods for detecting abnormalities using diagnostic planes have been proposed [19,35]. However, these approaches require many images from hundreds of CHD cases to develop a system to detect any CHDs, including rare CHDs. In addition, there are different and diverse forms, even within a given type of CHD. In contrast, there was no structural difference in the normal fetal heart; any deviation from the normal structure increases the possibility of CHD. Hence, to detect many types of abnormalities, our proposed method employs abnormality detection technology to detect deviations from the normal structure. In addition, the conventional screening procedure in the clinical field requires the determination of the plane that contributes to the diagnosis and to record images. However, this task is difficult to perform for unskilled examiners (especially in CHD); therefore, identifying diagnostic planes requires a high level of skill, closely related to that of diagnosis. To address this issue, we focused on the ultrasound video, which was obtained by scanning the entire fetal heart containing diagnostic planes. Deep learning-based abnormality detection methods for videos have been studied [68,69,70]; nonetheless, these methods were designed for surveillance videos and exhibit poor performance for videos with moving backgrounds [46]. Komatsu et al. proposed an abnormality detection method for fetal cardiac ultrasound screening videos. They used the sequential 20 video frames around diagnostic planes to calculate the abnormality scores [46]. Our proposed method utilizes the entire video frames, and the calculation of the abnormality score does not require any preprocessing, such as specifying the diagnostic planes. Therefore, the proposed graph chart diagrams and abnormality scores are highly applicable to fetal cardiac ultrasound screening. Moreover, we consider the potential for the further development of AI technology in fetal cardiac ultrasound. The graph chart diagram and calculated abnormality score can be used only for screening. They cannot be used for further diagnoses, such as identifying the type of CHD simultaneously. Analyses of various metrics must be considered using image segmentation [71,72,73] and other methods to effectively support the analysis of anomalies [35].

Our study demonstrates that graph chart diagrams improve abnormality detection by examiners with a wide range of experience, from experts and residents. Considering the residents, the mean AUC of the ROC curve with AI assistance was 0.748, which was not as high as 0.861 of AI. Detection via AI only may perform better than collaboration with examiners with low experience. This result indicates that a lower experience makes it more difficult to decide how to refer to AI information. Furthermore, considering the great performance improvement of residents using AI, the proposed methods can be used as educational and training tools. Regarding the fellows, the mean AUC increased from 0.829 to 0.890 when using AI. Their recall increased by 0.045 (=0.825−0.780), and their precision increased from 0.047 (=0.819−0.772) as shown in Table 3. Considering all the examiners, recall and precision increased by 0.059 (=0.822−0.763) and 0.066 (=0.827−0.761), respectively. Thus, fellows tended to place slightly more weightage on recall than on precision. Because the purpose of fetal cardiac ultrasound screening is not to miss CHDs, fellows, who are the main force in obstetrics, may place more importance on recall. Furthermore, AI usage improved the performance of experts from 0.966 to 0.975 in the mean AUC of the ROC curve. Regarding the experts, the increase in precision 0.015 (=0.927−0.912) was greater than the increase in recall, which was 0.007 (=0.963−0.956). This is probably because experts, who are estimated to be less than 5% of fellows in Japan, are required to make secondary judgments on cases classified by fellows. Therefore, they used AI to improve precision rather than recall. We found that fellows and experts can make good use of AI based on their respective roles, with fellows focusing on recall and experts on precision. For the residents, they achieved improvement in their performances with AI assistance; however, they could not achieve the performance obtained only by AI. This result implies that the examiners need experience in order to understand the explanations of explainable AI.

There are several limitations to this study. First, our proposed graph chart diagram is robust to probe movement; however, it has not been tested and evaluated for the influence of acoustic shadows in ultrasound videos. We may have to consider preprocessing, such as shadow detection [74]. Second, owing to the low incidence of CHD, we used a limited number of abnormal cases to test our proposed method. Furthermore, we mainly targeted severe CHDs and have not yet tested this method for the detection of mild abnormalities, such as small ventricular septal defects. Multicenter joint research is considerable to collect further CHD data for the validity and reliability evaluation of our explainable AI technology in future studies. Third, although the method is robust to probe movement, return, and speed, the robustness between devices has not been evaluated because of the limited number of ultrasound devices used for training. Finally, all training, validation, and test data in this study were acquired using the same type of ultrasound machine, and we did not perform the experiments on other machines. The generalization performance of the explainable AI that we have proposed is a subject for future studies.

## 5. Conclusions

We have proposed a graph chart diagram as an explainable AI technology for fetal cardiac ultrasound screening videos. This graph chart diagram exhibited a massive enhancement of screening performance in use by examiners of all experience levels. Furthermore, we showed that skilled examiners can improve their performance by appropriately using explainable AI for their respective roles. We also showed that less skilled examiners perform better with AI assistance than by themselves; however, they could not perform better than using only AI. Our study suggests that an examiner’s expertise will still be a key factor in medical examinations in the future with the widespread use of AI assistance. To address this point, we should consider the educational process to maximize the benefit of our technology. The progress of graphical user interface (GUI) technology has also the potential to improve AI assistance. We hope that explainable AI with these enhancements will enable support examiners with a wide range of experience and augment their medical professional capabilities towards the benefit of the patients.

## Figures and Tables

**Table 1:** Performance improvement by two techniques, cascade graph encoder and view-proxy loss.

Cascade Graph Encoder	View-Proxy Loss	Mean (SD)	Median (Min–Max)
√	√	0.861 (0.003)	0.860 (0.858–0.865)
√		0.819 (0.013)	0.813 (0.803–0.835)
	√	0.833 (0.002)	0.833 (0.830–0.835)
		0.798 (0.007)	0.803 (0.790–0.805)

The experiment was conducted five times for each combination. The abnormality score was calculated, and the AUC of the ROC curve was calculated for each trial. The table shows the mean (standard deviation) and median (minimum-maximum) values for each combination. ROC, receiver operating characteristic; AUC, area under curve; SD, standard deviation.

**Table 2:** Improvement in examiner performance using AI.

Method	Mean (SD)	Median (Min–Max)
Expert (n=8)	0.966 (0.017)	0.962(0.950–0.993)
Expert + AI (n=8)	0.975 (0.022)	0.981(0.945–1.000)
Fellow (n=10)	0.829 (0.084)	0.852(0.675–0.928)
Fellow + AI (n=10)	0.890 (0.037)	0.899(0.834–0.950)
Resident (n=9)	0.616 (0.128)	0.634(0.369–0.773)
Resident + AI (n=9)	0.748 (0.106)	0.746(0.474–0.836)
Examiner (n=27)	0.798 (0.167)	0.851(0.369–0.993)
Examiner + AI (n=27)	0.868 (0.113)	0.895(0.474–1.000)

This table shows the mean (standard deviation) and median (minimum-maximum) of the AUC of the ROC curves. Here, *n* denotes the number of cases. ROC, receiver operating characteristic; AUC, area under the curve; SD, standard deviation.

**Table 3:** Performance improvement in examiner decisions using AI.

Method	Accuracy (SD)	FPR (SD)	Recall (SD)	Precision (SD)	F1 Score (SD)
Expert (n=8)	0.928 (0.029)	0.100 (0.073)	0.956 (0.039)	0.912 (0.067)	0.934 (0.031)
Expert + AI (n=8)	0.938 (0.022)	0.081 (0.066)	0.963 (0.033)	0.927 (0.056)	0.943 (0.022)
Fellow (n=10)	0.775 (0.067)	0.230 (0.068)	0.780 (0.105)	0.772 (0.062)	0.774 (0.074)
Fellow + AI (n=10)	0.823 (0.036)	0.180 (0.071)	0.825 (0.072)	0.819 (0.051)	0.822 (0.038)
Resident (n=9)	0.603 (0.112)	0.356 (0.109)	0.572 (0.146)	0.616 (0.119)	0.589 (0.123)
Resident + AI (n=9)	0.731 (0.114)	0.233 (0.103)	0.694 (0.154)	0.746 (0.120)	0.716 (0.129)
Examiners (n=27)	0.763 (0.151)	0.233 (0.133)	0.763 (0.187)	0.761 (0.146)	0.760 (0.162)
Examiners + AI (n=27)	0.826 (0.108)	0.169 (0.102)	0.822 (0.146)	0.827 (0.111)	0.822 (0.119)

This table shows the accuracy, FPR, recall, precision, and F1 score with standard deviation for a certain value of the threshold. Here, n denotes the number of cases. We used 0.5 as a threshold. FPR, false positive rate; SD, standard deviation.

**Figure A1:**
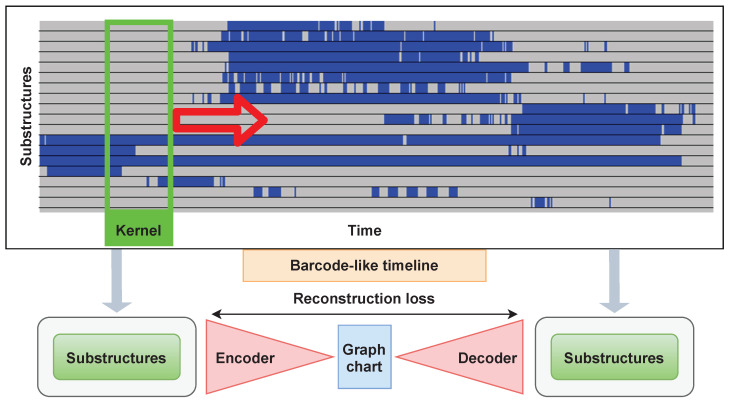
Process to compute a graph chart diagram with a simple auto-encoder. Kernel K slides over the barcode-like timeline in the time direction (red open arrow) and slices the detection status of the required substructure. The substructure detection status is input to the auto-encoder and compressed into two dimensions.

## Data Availability

For privacy reasons, we are unable to provide video data at this time. The source code of the proposed method, graph chart diagrams for one trial, and a small sample of barcode-like timeline data are provided at https://github.com/rafcc/2021-explainable-ai (accessed on 4 January 2022).

## Supplementary Materials

The following are available at https://www.mdpi.com/article/10.3390/biomedicines10030551/s1, Figure S1: Shapes created from the main-graph chart diagrams, Table S1: Cases of congenital heart disease and the gestational week at the time of acquisition, Video S1: A normal case, Video S2: A TOF case.

## Abbreviations

    The following abbreviations are used in this manuscript:

AI	artificial intelligence
AUC	area under the curve
CHD	congenital heart disease
DNN	deep neural network
FPR	false-positive rate
GE	General Electronic
Grad-CAM	Gradient-weighted Class Activation Mapping
ROC	receiver operating characteristic
SD	standard deviation
TOF	tetralogy of Fallot
TPR	true-positive rate
USA	United States of America
YOLO	You Only Look Once
3VTV	three-vessel trachea view
4CV	four-chamber view

## Appendix A. Formula of the Proposed Method

### Appendix A.1. Simple Auto-Encoder for Graph Chart Diagram

In this section, we elaborate on how to construct a graph chart diagram from a barcode-like timeline [46] using a simple auto-encoder. Barcode-like timelines are dependent on the speed of probe movement and are not robust to the inappropriate probe movement [46] as explained in Section 2.2.1. Considering a graph chart diagram, the simple auto-encoder (Figure A1) compresses a barcode-like timeline to obtain a time-independent representation that addresses the problem of barcode-like timelines; the fetal cardiac ultrasound videos (e.g., videos that do not start at a correct plane and move back and forth) are converted to this time independent expression. The one instance of a barcode-like timeline is denoted by $B\left(R\right)=\{b_{\left(r,t\right)}|b_{\left(r,t\right)}\in \left\{0,1\right\}$, $\left(r,t\right)\in R\times T_{B}\}$, where *R* is the set of substructures and $T_{B}$ is the set of times for all the frames. If the substructure *r* is detected at frame *t*, $b_{\left(r,t\right)}$ becomes one; otherwise, it becomes zero. We extract this barcode-like timeline with kernel size *w* and stride *s*. K(i,B(R)) is the set of $\{k_{\left(r,t\right)}|k_{\left(r,t\right)}\in B\left(R\right)$, (i−1)s<t≤(i−1)s+w}, which is a subset of the barcode-like timeline B(R). We also define K(0,B(R))=B(R) for i=0, which is used in Equation (A8). Here, we denote the encoder of the auto-encoder as E, the decoder as D, and the network weights of each as $\theta_{E}$ and $\theta_{D}$, respectively. Each point $g^{i}$ in the graph chart diagram is represented by $g^{i}\left(B\left(R\right);\theta_{E}\right)=E\left(K\left(i,B\left(R\right)\right);\theta_{E}\right)$. The reconstruction loss for a simple auto-encoder is calculated for each kernel and is expressed as

(A1) $$l_{\mathrm{rec}}\left(i,B\left(R\right);\theta_{E},\theta_{D}\right)=\mathrm{Loss}(K\left(i,B\left(R\right)\right),D\left(E\left(K\left(i,B\left(R\right)\right);\theta_{E}\right);\theta_{D}\right).$$

In this study, we adopted the binary cross-entropy for each element as the loss function.

The graph chart diagram is represented by $G=(g^{1},g^{2},\ldots ,g^{I+1})$, where *I* is the largest integer less than or equal to $(T_{\max}\left(B\right)-w)/s$; $T_{\max}\left(B\right)$ is the max time of the barcode-like timeline *B*.

### Appendix A.2. View-Proxy Loss

We can use the view-proxy loss to stabilize training by preventing the generation of “tangled” graph chart diagrams (Figure 2a). The view-proxy loss considers the ideal diagnostic plane (generally a “diagnostic plane” is known as “view”) as the proxy data. The view-proxy loss measures the distance between the points corresponding to automatically synthesized barcode-like timelines for the corresponding ideal diagnostic plane and the proxy data. Optimizing this loss stabilizes the formation of graph chart diagrams (Figure 2). We define a set of ${\tilde{B}}_{x}\left(R_{\mathrm{all}}\right)=\left\{{\tilde{b}}_{\left(r,t\right)}|{\tilde{b}}_{\left(r,t\right)}\in \left[0,1\right],\left(r,t\right)\in R_{\mathrm{all}}\times W\right\}$, which represents a synthesized barcode-like timeline corresponding to the ideal diagnostic plane and fixes the vector ${\tilde{g}}_{x}$ of a point on the graph chart diagram corresponding to ${\tilde{B}}_{x}$, where *W* is the set of the integer number from 1 to *w*, *x* denotes the diagnostic plane indicator. We also define that ${\tilde{R}}_{x}$ is the set of substructures and *y* denotes another diagnostic plane indicator. The view-proxy loss is expressed by the following equation.

(A2) $$\begin{array}{c} l_{\mathrm{vpl}}\left({\tilde{B}}_{x};\theta_{E}\right)=\mathrm{Loss}\left(\tilde{g_{x}},E\left({\tilde{B}}_{x}\left(R_{\mathrm{all}}\right);\theta_{E}\right)\right),\;\;{\tilde{b}}_{\left(r,t\right)}\in {\tilde{B}}_{x}\left(R_{\mathrm{all}}\right),\;\;{\tilde{b}}_{\left(r,t\right)}=\left\{\begin{array}{c} 1\;\;\;(r\in {\tilde{R}}_{x})\\ 0\;\;(r\in \tilde{R_{y}},y\neq x)\\ \mathrm{Uniform}\;\mathrm{random}\;\left[0,1\right]\;\;\;(r\notin \left({\tilde{R}}_{x}\cup {\tilde{R}}_{y}\right),y\neq x) \end{array}\right.. \end{array}$$

In this study, we used x,y∈{heart,vessels}, where ${\tilde{R}}_{\mathrm{heart}}$ and ${\tilde{R}}_{\mathrm{vessels}}$ denote substructures appearing especially in the ideal 4CV and 3VTV, respectively. For the loss function, we adopted the mean squared loss in all experiments. Considering the substructures, ${\tilde{R}}_{\mathrm{heart}}$ is the substructure that appears in the normal 4CV, indicating that ${\tilde{R}}_{\mathrm{heart}}=\{$crux, ventricular septum, right atrium, tricuspid valve, right ventricle, left atrium, mitral valve, left ventricle}. Thereafter, we applied ${\tilde{g}}_{\mathrm{heart}}=\left(0,1\right)$ as a corresponding vector on the graph chart diagram. Regarding the large vessels, ${\tilde{R}}_{\mathrm{vessels}}$ are the substructures that appear in the normal 3VTV, indicating that ${\tilde{R}}_{\mathrm{vessels}}=\{$pulmonary artery, ascending aorta, superior vena cava, ductus arteriosus}. Subsequently, we applied ${\tilde{g}}_{\mathrm{vessels}}=\left(1,0\right)$ as a corresponding vector on the graph chart diagram.

### Appendix A.3. Cascade Graph Encoder

The cascade graph encoder improves explainability by introducing prior knowledge of fetal cardiac substructures to the neural architecture of the auto-encoder. The cascade graph encoder achieves it by making a sub-graph chart diagram for each $R_{\mathrm{heart}}$ and $R_{\mathrm{vessels}}$ and making the main-graph chart diagram (Figure 1). This neural architecture allows us to see the contribution of each set of substructures to the main-graph chart diagram (Figure 4). Sub-graph chart diagrams are likely useful for further exploration of the decision process of the proposed method. The network weights of the encoder and decoder corresponding to the heart $R_{\mathrm{heart}}$ and vessels $R_{\mathrm{vessels}}$ of the auto-encoder that makes the sub-graph chart diagram are represented by $\theta_{E_{\mathrm{heart}}}$, $\theta_{E_{\mathrm{vessels}}}$, $\theta_{D_{\mathrm{heart}}}$, and $\theta_{D_{\mathrm{vessels}}}$, respectively. The network weights of the auto-encoder in the main-graph chart diagram are denoted by $\theta_{E_{\mathrm{main}}}$ and $\theta_{D_{\mathrm{main}}}$. The networks for the sub-graph chart diagrams are denoted as $E_{x}$ for the encoder (x={heart,vessels,others}) and $D_{x}$ for the decoder (x={heart,vessels}). The networks for the main-graph chart diagram are denoted as $E_{\mathrm{main}}$ and $D_{\mathrm{main}}$. Considering the substructures $R_{\mathrm{others}}=R_{\mathrm{all}}\backslash \left(R_{\mathrm{heart}}\cup R_{\mathrm{vessels}}\right)$, we use only the encoder and its network weights $\left(\theta_{E_{\mathrm{others}}}\right)$. We also define $\theta_{E_{\mathrm{sub}}}=\theta_{E_{\mathrm{heart}}}\cup \theta_{E_{\mathrm{vessels}}}\cup \theta_{E_{\mathrm{others}}}$, $\theta_{D_{\mathrm{sub}}}=\theta_{D_{\mathrm{heart}}}\cup \theta_{D_{\mathrm{vessels}}}$, $\theta_{E_{\mathrm{all}}}=\theta_{E_{\mathrm{sub}}}\cup \theta_{E_{\mathrm{main}}}$, and $\theta_{D_{\mathrm{all}}}=\theta_{D_{\mathrm{sub}}}\cup \theta_{D_{\mathrm{main}}}$. Each point in the main-graph chart diagram $g_{\mathrm{main}}^{i}$ is represented by the following equation.

(A3) $$g_{\mathrm{main}}^{i}\left(B;\theta_{E_{\mathrm{sub}}},\theta_{E_{\mathrm{main}}}\right)=E_{\mathrm{main}}\left(v;\theta_{E_{\mathrm{main}}}\right),$$

where

(A4) $$\left\{ \begin{array}{l} v=\left(g_{\mathrm{heart}}^{i}\left(B\left(R_{\mathrm{heart}}\right);\theta_{E_{\mathrm{heart}}}\right),g_{\mathrm{vessels}}^{i}\left(B\left(R_{\mathrm{vessels}}\right);\theta_{E_{\mathrm{vessels}}}\right),E_{\mathrm{others}}\left(K\left(i,B\left(R_{\mathrm{others}}\right)\right);\theta_{E_{\mathrm{others}}}\right)\right)\;\;\;\;\;\;\;\;\;\;\;\;\;\;\\ g_{\mathrm{heart}}^{i}\left(B\left(R_{\mathrm{heart}}\right);\theta_{E_{\mathrm{heart}}}\right)=E_{\mathrm{heart}}\left(K\left(i,B\left(R_{\mathrm{heart}}\right)\right);\theta_{E_{\mathrm{heart}}}\right)\\ g_{\mathrm{vessels}}^{i}\left(B\left(R_{\mathrm{vessels}}\right);\theta_{E_{\mathrm{vessels}}}\right)=E_{\mathrm{vessels}}\left(K\left(i,B\left(R_{\mathrm{vessels}}\right)\right);\theta_{E_{\mathrm{vessels}}}\right). \end{array}\right.$$

The loss function for the auto-encoder of main-graph chart diagram is

(A5) $$l_{\mathrm{main}}\left(i,B;\theta_{E_{\mathrm{all}}},\theta_{D_{\mathrm{all}}}\right)=\mathrm{Loss}\left(D_{\mathrm{main}}\left(g_{\mathrm{main}}^{i}\left(B;\theta_{E_{\mathrm{sub}}},\theta_{E_{\mathrm{main}}}\right);\theta_{D_{\mathrm{main}}}\right),K\left(i,B\left(R_{\mathrm{all}}\right)\right)\right).$$

We optimize the following formula for the *i*-th slice of the barcode-like timeline *B*:

(A6) $$\underset{\theta_{E_{\mathrm{all}}},\theta_{D_{\mathrm{all}}}}{\mathrm{minimize}}\;\;l_{\mathrm{main}}\left(i,B;\theta_{E_{\mathrm{all}}},\theta_{D_{\mathrm{all}}}\right)+\sum_{R,\theta }l_{\mathrm{rec}}\left(i,B\left(R\right);\theta \right),$$

where

(A7) $$\begin{array}{cc} \sum_{R,\theta }l_{\mathrm{rec}}\left(i,B\left(R\right);\theta \right) & =l_{\mathrm{rec}}\left(i,B\left(R_{\mathrm{heart}}\right);\theta_{E_{\mathrm{heart}}},\theta_{D_{\mathrm{heart}}}\right)+l_{\mathrm{rec}}\left(i,B\left(R_{\mathrm{vessels}}\right);\theta_{E_{\mathrm{vessels}}},\theta_{D_{\mathrm{vessels}}}\right). \end{array}$$

We denote one iteration of the optimization of Equation (A7) as the normal training step. Moreover, we perform regularization training steps for heart and vessels by adding view-proxy loss (Equations (A2)–(A7)):

(A8) $$\underset{\theta_{E_{\mathrm{all}}},\theta_{D_{\mathrm{all}}}}{\mathrm{minimize}}\;\;l_{\mathrm{main}}\left(0,{\tilde{B}}_{x};\theta_{E_{\mathrm{all}}},\theta_{D_{\mathrm{all}}}\right)+\sum_{R,\theta }l_{\mathrm{rec}}\left(0,{\tilde{B}}_{x}\left(R\right);\theta \right)+l_{\mathrm{vpl}}\left({\tilde{B}}_{x};\theta_{E_{\mathrm{all}}}\right),$$

where x∈{vessels,heart}. For $l_{\mathrm{vpl}}\left({\tilde{B}}_{x};\theta_{E}\right)$, we substitute Equation (A3) to the formulation of the view-proxy loss (Equation (A2)):

(A9) $$l_{\mathrm{vpl}}\left({\tilde{B}}_{x};\theta_{E_{\mathrm{all}}}\right)=\mathrm{Loss}\left(\tilde{g_{x}},g_{\mathrm{main}}^{0}\left({\tilde{B}}_{x};\theta_{E_{\mathrm{sub}}},\theta_{E_{\mathrm{main}}}\right)\right).$$

Considering the training process, three training steps (one for Equation (A7) and two for Equation (A8)) are performed alternately. In this study, we employed AdaGrad as the optimizer. For every ten normal training steps, we performed one regularization training step for the vessels and one for the heart. For more details, Algorithm A1 shows the process of generating graph chart diagrams, and we can see that a main-graph chart diagram is created after creating sub-graph chart diagrams. Algorithm A2 shows the training process, which consists of a normal training step, followed by regularization-training steps.

**Algorithm A1** Inference algorithm of the cascade graph encoder.
1: **Input:** Barcode-Like Timelines *B*; Stride Size *s*; Kernel Size *w*; Network Weights $\theta_{E_{\mathrm{all}}}$; Set of Substructures $R_{\mathrm{heart}}$, $R_{\mathrm{vessels}}$, $R_{\mathrm{others}}$ 2: **Output:** Sub-graph Chart Diagrams $G_{\mathrm{heart}}$, $G_{\mathrm{vessels}}$; Main-graph Chart Diagram $G_{\mathrm{main}}$ 3: i=1 4: **while**$\left(i-1\right)s+w\leq T_{\max}\left(B\right)$**do** 5: $g_{\mathrm{heart}}^{i}\left(B\left(R_{\mathrm{heart}}\right);\theta_{E_{\mathrm{heart}}}\right)=E_{\mathrm{heart}}\left(K\left(i,B\left(R_{\mathrm{heart}}\right)\right);\theta_{E_{\mathrm{heart}}}\right)$ 6: $g_{\mathrm{vessels}}^{i}\left(B\left(R_{\mathrm{vessels}}\right);\theta_{E_{\mathrm{vessels}}}\right)=E_{\mathrm{vessels}}\left(K\left(i,B\left(R_{\mathrm{vessels}}\right)\right);\theta_{E_{\mathrm{vessels}}}\right)$ 7: $G_{\mathrm{heart}}$⟵ add point $g_{\mathrm{heart}}^{i}\left(B\left(R_{\mathrm{heart}}\right);\theta_{E_{\mathrm{heart}}}\right)$ 8: $G_{\mathrm{vessels}}$⟵ add point $g_{\mathrm{vessel}s}^{i}\left(B\left(R_{\mathrm{vessels}}\right);\theta_{E_{\mathrm{vessels}}}\right)$ 9: $v=\left(g_{\mathrm{heart}}^{i}\left(B\left(R_{\mathrm{heart}}\right);\theta_{E_{\mathrm{heart}}}\right),g_{\mathrm{vessels}}^{i}\left(B\left(R_{\mathrm{vessels}}\right);\theta_{E_{\mathrm{vessels}}}\right),E_{\mathrm{others}}\left(K\left(i,B\left(R_{\mathrm{others}}\right)\right);\theta_{E_{\mathrm{others}}}\right)\right)$ 10: $g_{\mathrm{main}}^{i}\left(B;\theta_{E_{\mathrm{sub}}},\theta_{E_{\mathrm{main}}}\right)=E_{\mathrm{main}}\left(v;\theta_{E_{\mathrm{main}}}\right)$ 11: $G_{\mathrm{main}}$⟵ add point $g_{\mathrm{main}}^{i}\left(B;\theta_{E_{\mathrm{sub}}},\theta_{E_{\mathrm{main}}}\right)$ 12: **end while**

**Algorithm A2** Training algorithm of the cascade graph encoder.
1: **Input:** Set of Barcode-like Timelines {B}; Stride Size *s*; Kernel Size *w*; Max Iteration $n_{\max}$ 2: **Output:** Network Weights $\theta_{E_{\mathrm{all}}}$, $\theta_{D_{\mathrm{all}}}$ 3: Initialize network weights $\theta_{E_{\mathrm{all}}}$, $\theta_{D_{\mathrm{all}}}$ 4: n=0 5: **while** $n<n_{\max}$ **do** 6: *B*⟵ sample from {B} 7: i=1 8: **while** $\left(i-1\right)s+w\leq T_{\max}\left(B\right)$ **do** 9: Normal-training(i,B) 10: **if** *n* is a multiple of ten **then** 11: Synthesize ${\tilde{B}}_{\mathrm{heart}}$ and ${\tilde{B}}_{\mathrm{vessels}}$ 12: Regularization-training(${\tilde{B}}_{\mathrm{heart}},{\tilde{B}}_{\mathrm{vessels}}$) 13: **end if** 14: n=n+1 15: i=i+1 16: **end while** 17: **end while** 18: **procedure**Normal-training(i,B) 19: $\underset{\theta_{E_{\mathrm{all}}},\theta_{D_{\mathrm{all}}}}{\mathrm{minimize}}\;\;l_{\mathrm{main}}\left(i,B;\theta_{E_{\mathrm{all}}},\theta_{D_{\mathrm{all}}}\right)+\sum_{R,\theta }l_{\mathrm{rec}}\left(i,B\left(R\right);\theta \right)$ 20: **end procedure** 21: **procedure**Regularization-training(${\tilde{B}}_{\mathrm{heart}},{\tilde{B}}_{\mathrm{vessels}}$) 22: $\underset{\theta_{E_{\mathrm{all}}},\theta_{D_{\mathrm{all}}}}{\mathrm{minimize}}\;\;l_{\mathrm{main}}\left(0,{\tilde{B}}_{\mathrm{heart}};\theta_{E_{\mathrm{all}}},\theta_{D_{\mathrm{all}}}\right)+\sum_{R,\theta }l_{\mathrm{rec}}\left(0,{\tilde{B}}_{\mathrm{heart}}\left(R\right);\theta \right)+l_{\mathrm{vpl}}\left({\tilde{B}}_{\mathrm{heart}};\theta_{E_{\mathrm{all}}}\right)$ 23: $\underset{\theta_{E_{\mathrm{all}}},\theta_{D_{\mathrm{all}}}}{\mathrm{minimize}}\;\;l_{\mathrm{main}}\left(0,{\tilde{B}}_{\mathrm{vessels}};\theta_{E_{\mathrm{all}}},\theta_{D_{\mathrm{all}}}\right)+\sum_{R,\theta }l_{\mathrm{rec}}\left(0,{\tilde{B}}_{\mathrm{vessels}}\left(R\right);\theta \right)+l_{\mathrm{vpl}}\left({\tilde{B}}_{\mathrm{vessels}};\theta_{E_{\mathrm{all}}}\right)$ 24: **end procedure**

## Appendix B. Experimental Details

Firstly, we explain the configuration of the networks. The encoder $E_{x}$ flattens the input to a one-dimensional vector, followed by a fully connected layer with input $w|R_{x}|$ and output of two, and a sigmoid activation layer (x={heart,vessels,others}). The graph chart diagram is a two-dimensional diagram drawn in ${\left[0,1\right]}^{2}$, so the activation function is sigmoid, and the output of the encoder is set to two for every encoder. The decoder $D_{x}$ has a fully connected layer from two to $w|R_{x}|$ dimensions, followed by a sigmoid activation layer (x={heart,vessels}). The main encoder $E_{\mathrm{main}}$ has a fully connected layer with six to two dimensions, followed by a sigmoid activation layer. The main decoder $D_{\mathrm{main}}$ has a fully connected layer from two to the $w|R_{\mathrm{all}}|$ dimensions, followed by a sigmoid activation layer. All the network weights were initialized with a Gaussian distribution, and we used a different random seed for each trial. The kernel and stride sizes *w* and *s* were set to ten and five frames, respectively. Frames in which the kernel’s range extended beyond the barcode-like timeline were removed. Therefore, the last few frames of the video were not used for training or inference. The iterations were set to 100,000, which was sufficient for the training to converge. Additional optimization iteration with view-proxy loss was performed for every ten iterations as explained in Equation (A8)). Regarding the optimization, we used the AdaGrad optimizer with an initial learning rate and accumulator value of 0.7 and 0.1, respectively. Considering the optimization with view-proxy loss, we used another AdaGrad optimizer with an initial learning rate and accumulator value of 0.1 and 0.1, respectively. The mini-batch size was set to one for the limitation of our implementation. The network weights and hyperparameters used to calculate the barcode-like timeline are the same as those in Komatsu et al. [46] Window size *w*, stride size *s*, learning rate, and accumulator value were determined using a validation set introduced in the data preparation section. Considering the software version, Python version 3.7 was used. Tensorflow 1.14.0, scikit-learn 0.24.2, OpenCV-Python 4.5.1, NumPy 1.20.3, and Shapely 1.7.1 Python libraries were used. Regarding the hardware, we used a server with an Intel(R) Xeon(R) CPU E5-2690 v4 at 2.60 GHz with a GeForce GTX 1080 Ti.
