# Supplementary material for: Medical Professional Enhancement Using Explainable Artificial Intelligence in Fetal Cardiac Ultrasound Screening
Source: Biomedicines. 2022 Feb 25;10(3):551. doi: 10.3390/biomedicines10030551 (PMC8945208; doi:10.3390/biomedicines10030551)
Supplement: Supplementary file 1 [file biomedicines-10-00551-s001.zip › Biomedicines_supplementary/supplementary.pdf]

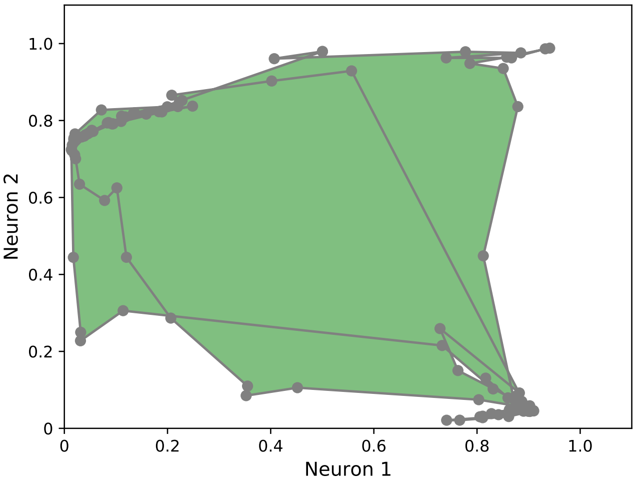

(a) Normal

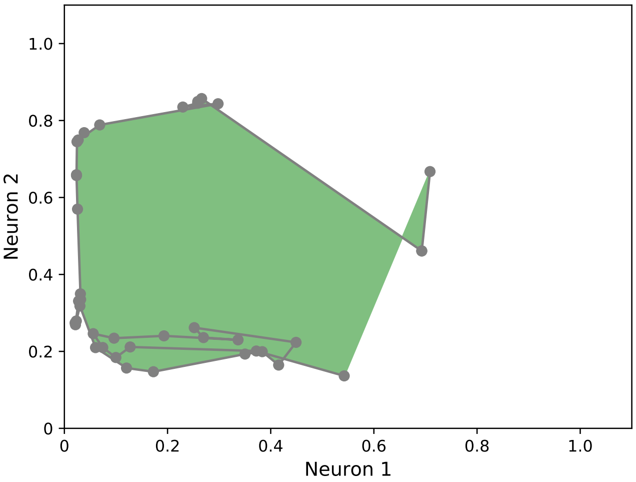

(b) Tetralogy of Fallot

Supplementary Figure S1. Shapes created from the main-graph chart diagrams. The green semitransparent areas represent the shapes detected using the Shapely package in Python for the graph chart diagram corresponding to Fig. 3 of the main script. The dots circled in gray correspond to each kernel, and they are connected as the kernel moves as shown in Fig. 3 in the main text. If the start and end points are not closed, similar to the tetralogy of Fallot, they are automatically connected by the library. The abnormality score $\Gamma_{\mathrm{AI}}(G)$ for normal cases is 0.056 (**a**), and that of $\Gamma_{\mathrm{AI}}(G)$ for the tetralogy of Fallot is 0.729 (**b**). Figure 3 and the abnormality score are shown to the examiner; however, the shape figures are not shown.

Supplementary Table S1. Cases of congenital heart disease and the gestational week at the time of acquisition.

Congenital heart disease (CHD)	Gestational week
Validation dataset	
AVSD	19
DORV-AVSD	21
AoCo-VSD	29
Test dataset	
TGA1	19
TGA1	19
TA	20
Ebstein	21
TOF	21
DORV-TGA-VSD	21
PAIVS	23
RAA	25
HLHS	28
TOF	29

AVSD, atrioventricular septal defect; DORV, double-outlet right ventricle; AoCo, coarctation of the aorta; VSD, ventricular septal defect; TGA1, transposition of the great arteries type-1; TA, tricuspid atresia; TOF, tetralogy of Fallot; PAIVS, pulmonary atresia with intact ventricular septum; RAA, right aortic arch; HLHS, hypoplastic left heart syndrome.
