# Supplementary figures and images for: Medical Professional Enhancement Using Explainable Artificial Intelligence in Fetal Cardiac Ultrasound Screening
Source: Biomedicines. 2022 Feb 25;10(3):551. doi: 10.3390/biomedicines10030551 (PMC8945208; doi:10.3390/biomedicines10030551)

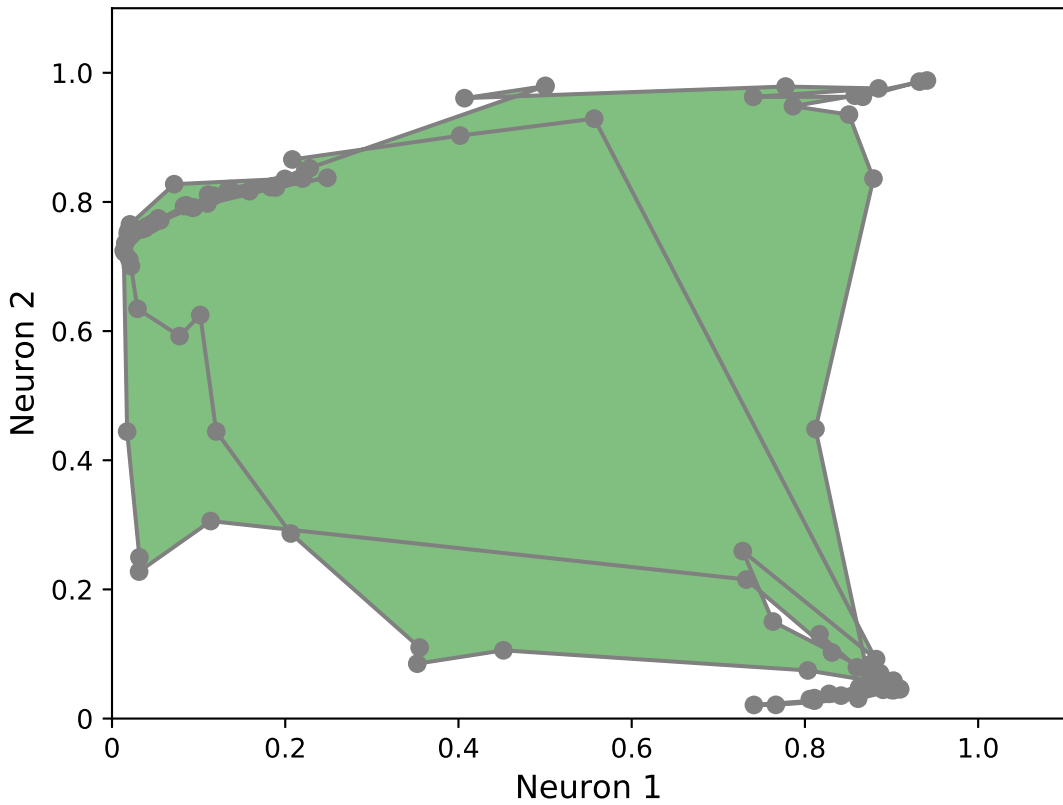

Supplement: Supplementary file 1 [file biomedicines-10-00551-s001.zip › Biomedicines_supplementary/appfigs/1062_fill.pdf]

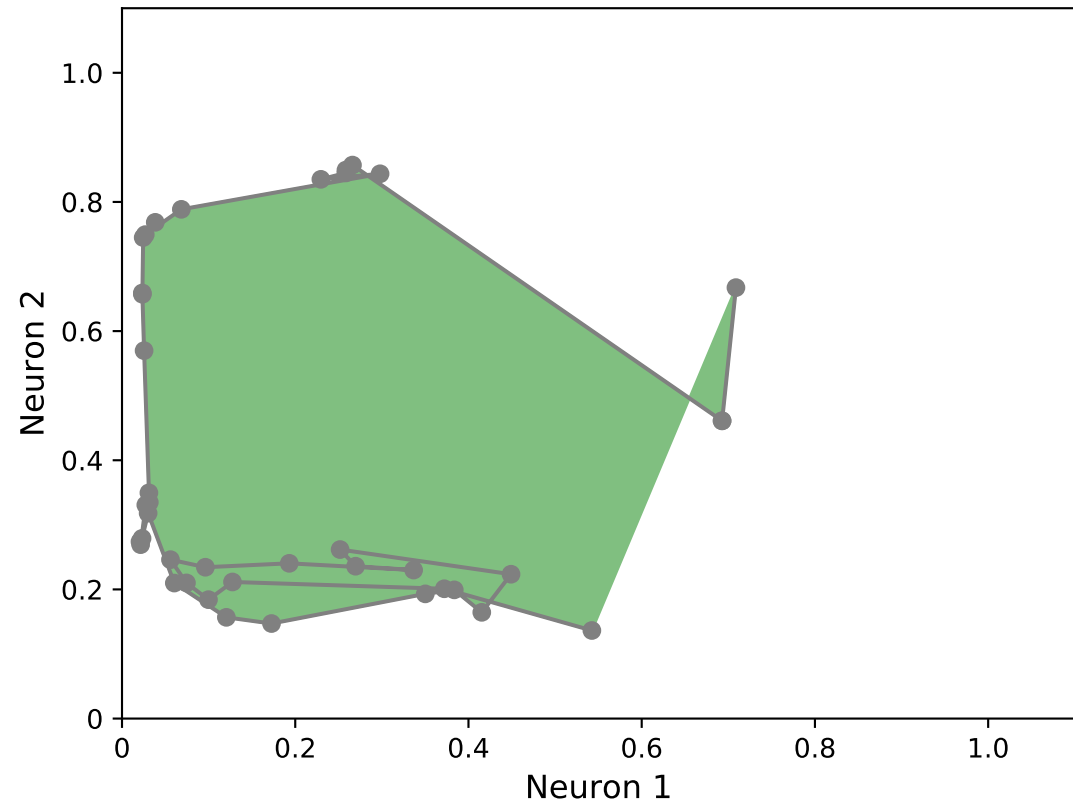

Supplement: Supplementary file 1 [file biomedicines-10-00551-s001.zip › Biomedicines_supplementary/appfigs/1_fill.pdf]
